# Dental Unit Waterlines: A Survey of Practices in Eastern France

**DOI:** 10.3390/ijerph16214242

**Published:** 2019-11-01

**Authors:** Alexandre Baudet, Julie Lizon, Jean-Marc Martrette, Frédéric Camelot, Arnaud Florentin, Céline Clément

**Affiliations:** 1 Faculty of Dentistry, University of Lorraine, 54505 Vandœuvre-lès-Nancy, France; jean-marc.martrette@univ-lorraine.fr (J.-M.M.); celine.clement@univ-lorraine.fr (C.C.); 2 Department of Dentistry, University Hospital, 54000 Nancy, France; 3 Department of Hygiene and Environmental Analysis, University Hospital, 54505 Vandœuvre-lès-Nancy, France; j.lizon@chru-nancy.fr (J.L.); arnaud.florentin@univ-lorraine.fr (A.F.); 4 EA 3450 DevAH, University of Lorraine, 54505 Vandœuvre-lès-Nancy, France; 5 Dental Private Practice, 88300 Neufchateau, France; frederic.camelot@wanadoo.fr; 6 Faculty of Medicine, University of Lorraine, 54505 Vandœuvre-lès-Nancy, France; 7 EA 4360 APEMAC, University of Lorraine, 54505 Vandœuvre-lès-Nancy, France

**Keywords:** water quality, infectious control, occupational practices, dental chair, waterlines

## Abstract

Water is essential during dental care. Physical and chemical techniques should be used to maintain a good water quality with respect to bacteria, and to ensure the safety of exposed patients and dental staff. The aim of this survey was to assess the modalities used by dental practitioners in Eastern France to maintain the water quality of their dental unit waterlines (DUWLs). A questionnaire about water quality maintenance practices was sent to 870 dental offices in 2016. The questionnaires were completed by 153 dental offices, covering about 223 dental care units. The majority of units were fed by mains water (91.0%), which is generally unfiltered (71.3%). One-third (33.6%) of the units had an independent water bottle reservoir. Flushing, a basic physical technique to improve the quality of units’ outflow water, was practiced in 65.4% of dental offices. Concerning the chemical treatment of water, it was used for 62.1% of the units. An analysis of the microbiological quality of the DUWL water was only carried out in 2.6% of the offices. In conclusion, providing better training to dental staff seems necessary to improve their practices and to generalize procedures that improve the microbiological quality of the water used.

## 1. Introduction

Water is an essential element involved in dental care, as it allows cooling and irrigation of certain parts of equipment such as rotors and mechanical scalers. Furthermore, it acts on treated teeth to avoid iatrogenic overheating, to remove debris from the surgical site, and to rinse the patient’s mouth. Although the water used in dental offices is not standardized, it must at least meet criteria of drinking water. The maintenance of water quality is crucial because patients and dental staff are exposed to water through three routes during dental care. Firstly, they are exposed to water by projection on skin and mucous membranes. Secondly, they are exposed to water by aerosol. Bio-aerosols generated during dental care contain micro-organisms [1,2] able to disperse to a distance of one meter around the patient and remain in suspension for twenty minutes [3]. Thirdly, patients can ingest water during dental care.

Dental unit waterlines (DUWLs) have an accumulating and growing biofilm on their inner surfaces. This serves as a reservoir of micro-organisms that will be released during dental care in the water circulating inside the DUWL [4]. Unfortunately, the tubes of dental units provide a favorable environment for the development of biofilms for many reasons. These are as follows: the quality of the water supplying the unit [4,5]; the numerous periods of inactivity of the unit accompanied by water stagnation [2,5]; the narrowness of the pipes, which generates a low water flow rate at the periphery of the canal lumen [5,6]; the complex interconnections of long and narrow waterlines of units [5,6]; the plastic materials constituting the tubes [7]; the anti-retraction valves that fail to completely prevent retro-contamination by oral fluids [8,9]; and sometimes the presence of a water heating device. This last produces a pleasant water temperature for the patient, but favors the development of micro-organisms [1,10].

The DUWL output water often contains multiple bacteria in greater quantities than recommended [1,2,11,12], including opportunistic pathogenic bacteria such as *Pseudomonas aeruginosa* [2,12], *Legionella pneumophila* [2,12,13,14], and nontuberculous *Mycobacterium* [15]. Only a few cases of infections related to dental units’ water have been reported [15,16]. However, the immunocompromised, cancer patients, diabetic, very young, and elderly patients are particularly vulnerable populations to contaminated water [17]. Some infections have been reported to be fatal for patients [13,14] and a possible fatal case was reported for one dentist [18]. Thus, while the incidence of reported dental unit waterborne infections appears to be minimal, the risk should not be underestimated. Some infections have probably not been identified because of a failure to associate infections with exposure to DUWL output water. Sporadic infections not requiring hospitalization are not likely to be investigated in depth, and it would be extremely difficult to trace the origin of an infection contracted from contaminated DUWL output water if clinical manifestations develop a number of weeks after exposure [4,19].

The water used in dentistry is not standardized, so it must only meet the criteria of drinking water. In France, that means the absence of *Escherichia coli* and *Enterococci*, with an ideal revivable aerobic flora at 22 °C less than 100 colony-forming units per milliliter (CFU/mL) at the inlet, and a differential of less than a factor of 10 between inlet and outlet water [17]. In the United States, the Center for Disease Control and Prevention (CDC) recommends less than 500 CFU/mL in water used for non-surgical dental care [20]. For surgical procedures, the use of sterile water is recommended [17,20]. The French dental guideline towards the prevention of healthcare-associated infections re-iterates that dentists must ensure that a set of physical and chemical measures are in place to maintain an acceptable water quality of their units’ outflow [17].

The aim of this survey was to assess the means used by dentists operating outside public health institutions in Eastern France to maintain the water quality of their dental care units.

## 2. Materials and Methods 

Our study dates from 2016. A cross-sectional study with self-administered questionnaires was conducted among dentists of Eastern France operating outside health care institutions. A letter explaining the context and some terms necessary for an understanding of the study was distributed with the questionnaire. The participants had the opportunity to contact a researcher (C.C.) in case of doubt during the administration of the questionnaire.

In our study, 870 questionnaires of practitioners’ knowledge and practices about dental unit water quality were sent to dental offices from a database created using the yearbooks of dentists and orthodontists. We asked dentists to complete these questionnaires and return them to us by mail.

The questionnaire was composed of two sections. The first section consisted of questions to report the dental equipment used at the dental office (units, implantology and surgical motors, ultrasound scaler generators) and the means used to guarantee the water quality (flushing, filter, water softening, existence of an independent bottle reservoir, disinfection protocol). The second section explored the characteristics of each dental chair present in the dental office (brand, model, age, maintenance, feed water, disinfection products).

Participation was voluntary and without any compensation. Anonymity was guaranteed at the phase of data analyses. Ethical approval was not required.

Regarding data analysis, blank questionnaires returned to us were excluded, whereas those that were totally or only partially completed were included in the study. The data were collected on Access^®^ (Microsoft Corporation, Redmond, WA, USA) and analyses were performed using EpiInfo^®^ 7.2.2.2. (CDC, Atlanta, GA, USA). 

## 3. Results

The questionnaire was completed and returned by 153 dental offices in Eastern France, representing a response rate of 17.6%.

In our study, 62.1% of dental offices only had one unit, 28.8% had two units, and 9.1% had three or more units. A total of 223 units of different brands (Figure S1) were identified in the 153 offices that participated in the study. Concerning units, at 4.5%, were of a different make from the dental chair. On average, dental chairs were 8.1 (±5.6) years old (68.3% were more than five years old), with a last maintenance date of less than 6 months (51.6%). Maintenance of the remaining chairs ranged from 6 months to 1 year (22.4%), and more than 1 year (19.3%). A quarter of the dental offices were equipped with implantology motors (25.5%) and 37.9% had ultrasound scaler generators independent of the unit.

**Figure 1:**
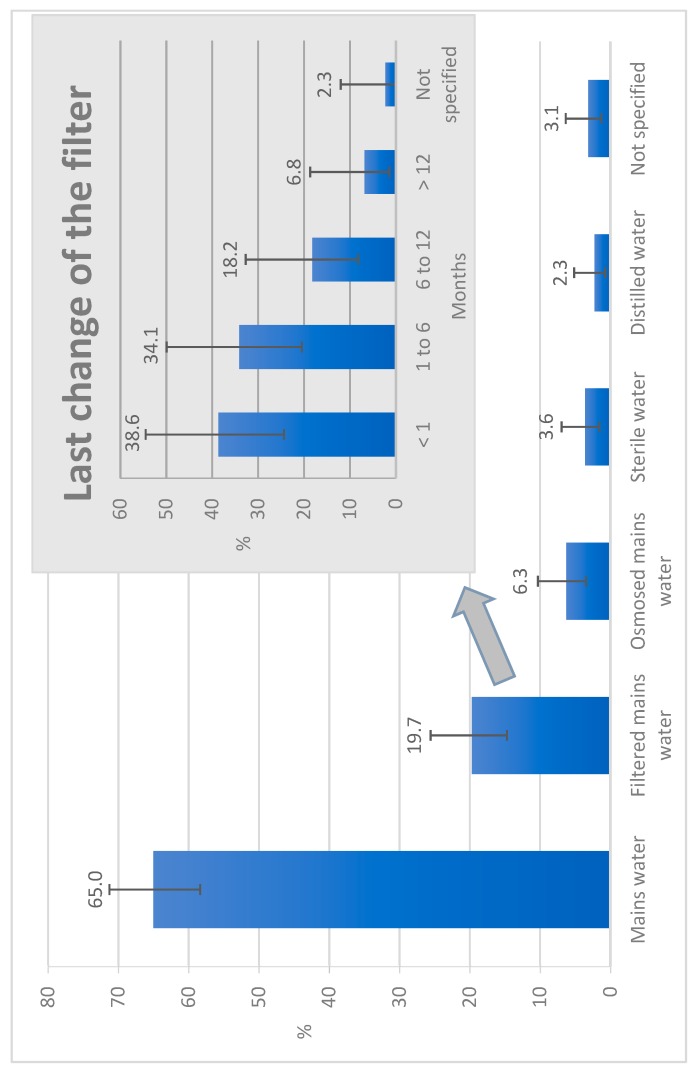
Water supply of the 223 dental care units studied in Eastern France in 2016. Note: Filtered water was passed through a 0.2-µm filter to remove impurities and bacteria; Osmosed water was purified through a semi-permeable membrane; Sterile water was free of any micro-organisms and toxins in reference to European pharmacopeia; Distilled water was purified after having been boiled into vapor and condensed back into liquid.

**Figure 2:**
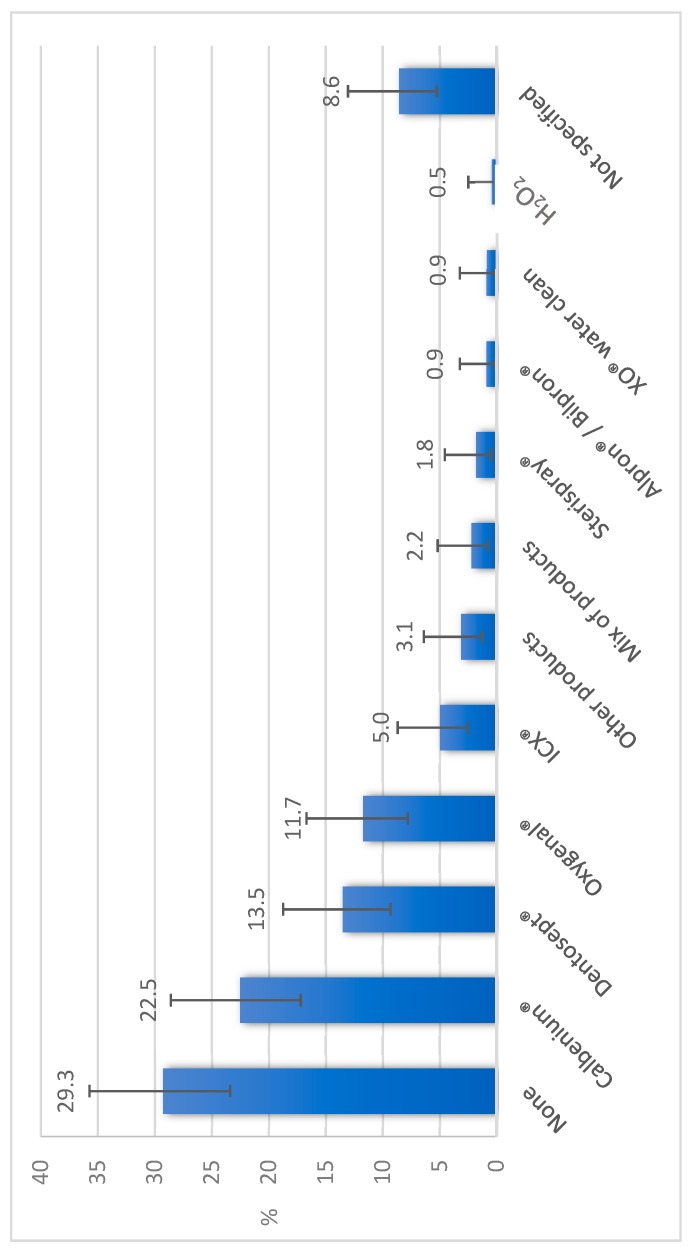
Products used for the chemical treatment of water in the 223 dental care units studied in Eastern France in 2016. Note: Calbenium^®^ contains quaternary ammonium, EDTA, and sodium tosylchloramide; Dentosept^®^, Oxygenal^®,^ and XO^®^ water cleaners are made of H_2_O_2_; ICX^®^ contains sodium percarbonate, silver nitrate, and cationic surfactants; Sterispray^®^ contains benzalkonium chloride, chloramine T, and EDTA; Alpron^®^/Bilpron^®^ contain EDTA and polyaminopropyl biguanide with sodium tosylchloramide for Alpron^®^ and with ester p-hydroxybenzoate for Bilpron^®^.

Concerning the water supply of dental offices, 28.8% were equipped with a water softener. Dental units were mainly (91.0%) supplied with mains water (Figure 1), with 26.0% of the units having an integrated tank and 33.6% an independent bottle reservoir. Flushing of the units was practiced in 65.4% of offices. Flushing was practiced at the beginning of the day in 57.5% of offices, between two patients in 22.2% of offices, and at the end of the day in 35.9% of offices (Table 1). When performed between two patients, dynamic instruments were left in place in 80.0% of cases. Chemical water treatment was implemented for 62.1% of the units, using various products (Figure 2). Concerning dental offices equipped with independent bottle reservoirs, 60.0% disinfected them with different frequencies (Table 2) and methods (Table 3), and a spare bottle was available in 55.6% of offices. All dentists who practice implantology activity reported using sterile water to perform this surgery, but if we look at all the practitioners, 51% did not use sterile water. Only 2.6% of offices had commissioned a microbiological analysis of the water in their structure, while 19.6% of practitioners thought that the microbiological quality of the water at the output of their units was identical to the water supplying it. Further, 63.4% of the dentists were afraid of developing an infection during their activity.

## 4. Discussion

In France, dental units are very often supplied with municipal water, which is chlorinated drinking water (0.2 mg/L). This was the case for 91.0% of the units in our study (65.0% were directly supplied by mains water, 19.7% were supplied by filtered mains water, and 6.3% were supplied by osmosed mains water). The water supply of the dental units varies from one country to another: in Europe mains water is used in 64% of dental offices, with major use in Germany, Greece, and the Netherlands [21]; while there is low use in the United Kingdom, where distilled water is preferred [21,22]. The mains water was filtered for 19.7% of the units in our study—a similar figure to the European average of 21% [21]. One-third (33.6%) of the DUWLs in our study had an independent water reservoir, which is better than the European average of 27% [21]. However, this is worse than in the USA, where 62% of practitioners have an independent water circuit [23] and in the United Kingdom, where about 95% of units have an independent bottle reservoir [21,22]. Bottle reservoirs allow the water to be cut off from the mains water, enable the choice of which type of water is used for the care (filtered mains water, distilled water, or sterile water), and to correctly determine the concentration of the chemical treatment chosen by the dentist to ensure better control of the microbiological quality of the unit’s water. However, handling of the bottle reservoir must be done with care not to contaminate the water with micro-organisms from the hand [19].

Two simple physical modalities significantly reduced the bacterial load in DUWLs. First, a 0.2-μm filter was placed in 19.7% of the units in our study. Second, flushing is performed at the beginning of the day to remove some of the micro-organisms from the biofilm present on the inner surfaces of the tubes, and between each patient to fight against retro-contamination. In our study, 65.4% of the dentists performed flushing, but only 57.5% of the chairs were flushed at the beginning of the day, and only 22.2% were flushed between each patient. These figures are less satisfactory than in other French departments where 91.5% of the dentists performed flushing once a day in 63% of cases, and between each patient for 19% [24]. In Europe, 49% of the units are flushed between patients, although there are significant variations from one country to another [21]. In England, 56% of the units are flushed at the beginning of the day and 29% between each patient [22]. In Scotland, where 40 chairs were studied, 62.5% were never flushed, only 5% were flushed daily, and 2.5% were flushed between each patient [11]. The Organization for Safety, Asepsis and Prevention (OSAP) recommended that DUWLs be flushed for 20–30 s at the beginning and end of each day, and after each patient to remove patient material potentially retracted during treatment [25].

In addition to physical modalities, chemical treatment of water also significantly reduces the quantity of micro-organisms at the outlet of the unit [5,26,27]. DUWL water can be disinfected by various types of chemicals—punctual or continuous treatments with one or two biocides. A couplet of biocides provides a differential treatment during the phases of unit activity and rest [28]. It appears that continuous water treatments are significantly more efficacious than punctual treatments [26,27]. In our study, a chemical treatment of water was practiced in 62.1% of the units, which is higher than generally practiced in Europe, where 45% of the dentists declared treating the water of their unit [21]. In Eastern England, 50% of the units are treated with disinfectants [22]. The highest figure was in another French department, where 88% of dentists chemically treated their water (71% of cases had continuous treatment of water either alone or in combination with a punctual treatment, and 21% of cases used punctual treatment only) [24].

The majority (68.3%) of the dental chairs used in our study were more than five years old—a little older than the European average [21]. Ji et al. showed a significant positive correlation between the age of the dental chairs and the retracted volumes of water in the DUWL due to wear and tear of the anti-retraction systems [9]. However, the age of dental chairs did not appear to influence the microbiological water quality [1,11].

To ensure microbiological control of the DUWL outflow, dentists could carry out a microbiological analysis of their water at least annually [29]. According to the CDC and the OSAP, this monitoring helps to ensure the effectiveness of procedures and permit the identification of failures in clinical water management practices [20,30]. However, in our study, only 2.6% of dentists had already done such an analysis. This figure is extremely low, and globally these analyses are not frequently practiced in other countries. In the USA, 16.8% of the dentists perform an annual bacteriological analysis of DUWL water [31]. In Europe, 17% of dentists perform water analysis and the German dentists are most involved at a rate of 70% [21]. It is important to note that the CDC, the OSAP, and the American Dental Association (ADA) recommend that periodic monitoring should be performed according to the manufacturer’s instructions [20,25,30].

Dentists’ knowledge of how to maintain the water quality of their units is sometimes wrong [32]. In our study, several dentists confused the chemical treatment of their unit’s feed water with the chemical treatment of the unit’s suction. In France, dentists’ knowledge about the risks associated with the water they use during care is rather low. Indeed, 37.2% of dentists say they have little or no knowledge on the subject, 68% believe that there is a risk of developing a biofilm in their unit, and only 28% think that the water in their unit may present a risk to their patients. In the dental care context, 90% of French dentists expressed the wish to be more informed about infectious risks related to water [24]. The same trend is seen on a European scale, where almost half of dentists believe that the quality of the DUWL outflow is the same as the DUWL inlet. Furthermore, 35% think that this water can be dangerous for them, 32% are anxious for their team, and 48% are anxious for their patients. In addition, 65% feel strongly concerned about the water quality of their unit; 89% would support regular microbiological analyses of their water, and a vast majority (98%) would be happy to accept simple advice on how to disinfect the DUWL water [21].

Dentists are responsible for the quality and safety of care. They must complete a continuous professional development program, including the prevention of health-care-associated infections. The CDC and the ADA recommend that dentists consult the manufacturer of their dental unit to determine the best method for cleaning DUWLs and maintaining acceptable water quality [20,30]. In practice, more than two-thirds of dentists (68%) maintain their units according to the manufacturer instructions [24]. So, manufacturers have an important role in controlling the risk of health-care-associated infections [33]. They must design dental care units and develop means to limit the risks of biofilm development. They must provide products and instructions to dental staff for the maintenance of dental care units [4,25]. 

## 5. Conclusions

Progress is necessary to control the microbiological quality of DUWLs. Our investigation indicates that the physical and chemical approaches to limiting the proliferation of bacterial water in DUWLs are insufficiently practiced. They require training and follow-up of the protocols by dental staff to ensure the safety of patients with respect to the infectious risks related to the water used during dental care. Guidelines to explain how to maintain the water quality of the dental care units have been made by the CDC, the OSAP, and the ADA, for example. Initial training and continuous professional development should be strengthened for dental staff. Moreover, it appears necessary to develop and validate standard protocols for maintaining and monitoring DUWLs.

This first French regional study about the ways used by dentists to maintain DUWL water quality requires further investigation to analyze the microbiological water quality of these DUWLs. 

## Figures and Tables

**Table 1:** Flushing times of the dental unit waterlines in 153 dental offices studied in Eastern France in 2016.

Flushing Opportunity	No Flushing		<20 Seconds		20 Seconds to 1 Minute		>1 Minute		Not Specified	
	*n*	%	*n*	%	*n*	%	*n*	%	*n*	%
At the beginning of the day	59	38.6	22	14.4	45	29.4	21	13.7	6	3.9
Between two patients	112	73.2	19	12.4	13	8.5	2	1.3	7	4.6
At the end of the day	92	60.2	6	3.9	21	13.7	28	18.3	6	3.9

**Table 2:** Disinfection frequency of the independent water bottle reservoirs in 36 dental offices equipped among 153 studied in Eastern France in 2016.

Every Day		More than Once a Week		Once a Week		Less than Once a Week	
*n*	%	*n*	%	*n*	%	*n*	%
9	25.0	7	19.4	15	41.7	5	13.9

**Table 3:** Disinfection methods of the independent water bottle reservoirs in 36 dental offices equipped among 153 studied in Eastern France in 2016.

Soap and Water		Sodium Hypochlorite		Sodium Hypochlorite + Thermal Washer-Disinfector		Thermal Washer-Disinfector		Other		Not Specified	
*n*	%	*n*	%	*n*	%	*n*	%	*n*	%	*n*	%
6	16.7	16	44.4	2	5.5	1	2.8	10	27.8	1	2.8

## Supplementary Materials

The following are available online at https://www.mdpi.com/1660-4601/16/21/4242/s1, Figure S1: Makes of the 223 dental care units studied in Eastern France in 2016.
