# Supplementary material for: Dental Unit Waterlines: A Survey of Practices in Eastern France
Source: Int J Environ Res Public Health. 2019 Nov 1;16(21):4242. doi: 10.3390/ijerph16214242 (PMC6862618; doi:10.3390/ijerph16214242)
Supplement: Supplementary file 1 [file ijerph-16-04242-s001.pdf]

International Journal of
Environmental Research and Public Health

MDPI

Supplementary Material: Dental Unit Waterlines: A Survey of Practices in Eastern France

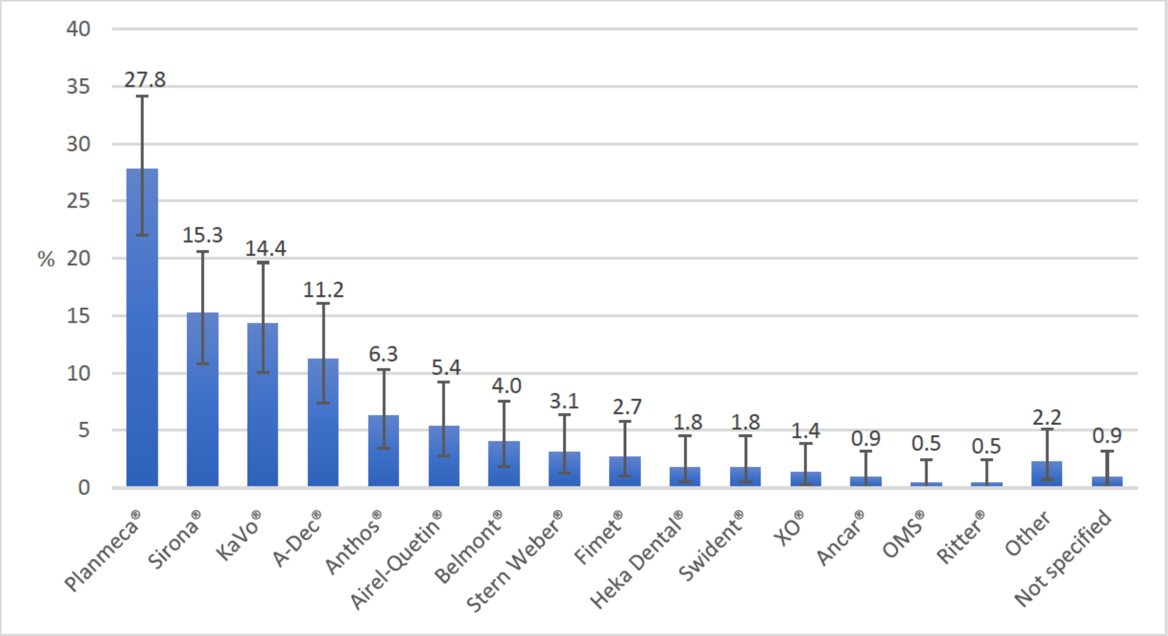

Figure S1. Brands of the 223 dental care units studied in Eastern France in 2016.
